# Use of Intrapleural Fibrinolytic Therapy in a Trapped Lung following Acute Traumatic Haemothorax

**DOI:** 10.1155/2021/5592086

**Published:** 2021-06-21

**Authors:** Chuan T. Foo, Jurgen Herre

**Affiliations:** Cambridge University Hospitals NHS Foundation Trust, Department of Respiratory Medicine, Cambridge, UK

## Abstract

Retained haemothorax is a common sequela of traumatic haemothorax and refers to blood that cannot be drained from the pleural cavity. We report a case of trapped lung secondary to retained haemothorax in a patient who sustained a penetrating chest injury. Initial chest computed tomography (CT) showed a large haemothorax that was managed with an intercostal drain insertion (ICD). Repeat chest CT and thoracic ultrasonography performed after ICD removal showed an organized pleural space resembling haematoma. ICD was reinserted with administration of intrapleural fibrinolytic therapy (IPFT). Subsequent chest CT showed the development of a pleural rind and trapped lung. A second ICD was inserted, and further IPFT were administered together with aggressive negative pressure suction. Haemoglobin remained stable. The patient made a full recovery and imaging performed two weeks later showed minor blunting of the costophrenic angle. This case highlights the feasibility and safety of IPFT in the management of trapped lung associated with traumatic retained haemothorax as an alternative to surgery.

## 1. Introduction

Haemothorax is an accumulation of blood in the pleural space and is often the result of trauma to the chest. Retained haemothorax refers to blood that cannot be drained from the pleural cavity and is associated with complications including empyema and fibrothorax. Traditionally, haemothorax is managed surgically with tube thoracostomy drainage and video-assisted thoracoscopy surgery. Despite the rise in the utilisation of intrapleural fibrinolytic therapy in parapneumonic effusions and empyema, its role in haemothorax remains unclear. We present a case of trapped lung associated with acute traumatic haemothorax complicated by retained haemothorax that was successfully managed with intrapleural fibrinolytic therapy, highlighting the feasibility and safety of this management strategy.

## 2. Case Report

A 19-year-old male was transferred from a local district hospital to our centre for the management of traumatic chest injuries. The patient had been involved in an altercation and sustained three knife stab injuries. Two of these were superficial and affected the right upper and lower chest wall. The third was a deep penetrating injury to the left hemithorax.

Chest computed tomography (CT) performed at the local district hospital showed a large volume left sided haemothorax which was managed with a 24Fr intercostal drain (ICD). As a temporizing measure, the penetrating injury was packed with a ribbon gauze, and after the patient was stabilized, he was transferred to our regional level 1 trauma centre. The patient received 9 units of packed red blood cells, 2 units of fresh frozen plasma, and 2 units of platelets prior to transfer.

**Figure 1:**
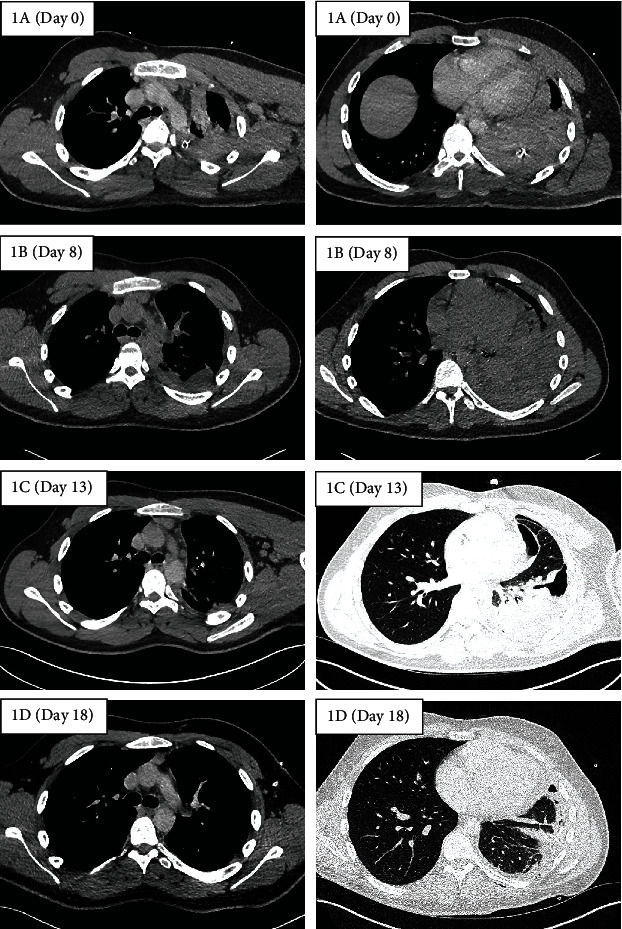
Serial axial chest computed tomography (CT) demonstrating reduction in size of the left sided haemothorax over time. Images on the left and right represent CT slices taken at the level of the carina and the lung base, respectively. Day 0 refers to day of admission.

On examination, heart rate was regular at 93 beats/min, blood pressure of 106/55 mmHg, respiratory rate of 34 breaths/min, and oxygen saturation of 97% on 2 L/min supplemental oxygen. The ICD had drained 1800 mL of blood, and the blood test showed a haemoglobin of 140 g/L (normal 135-172 g/L), platelet of 263 × 10^9^ (150-370 × 10^9^), prothrombin time of 13.4 s (10.8-13.3 s), activated partial thromboplastin time of 24.7 s (28.2-36.6 s), and fibrinogen of 2.62 g/L (1.46-3.33 g/L). Renal and liver function tests were within normal limits. Chest CT demonstrated a slight reduction in the size of the left sided haemothorax and no evidence of active bleeding (Figure 1(a)). The ICD was up sized to a 32Fr to promote clot removal, and prophylactic intravenous co-amoxiclav was administered. No further blood products were required, and the patient was managed conservatively.

**Figure 2:**
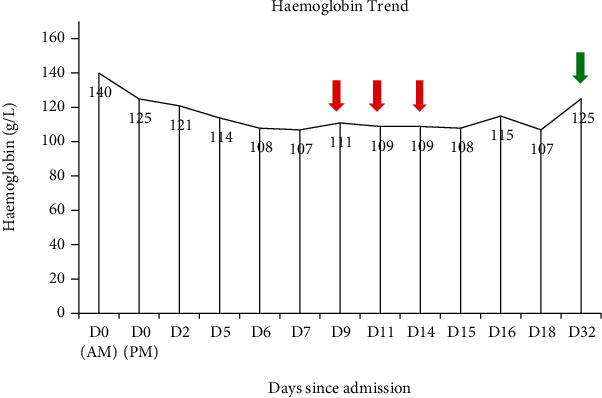
Haemoglobin trend throughout admission. The red arrow indicates administration of intrapleural fibrinolysis. The green arrow indicates haemoglobin on follow-up.

**Figure 3:**
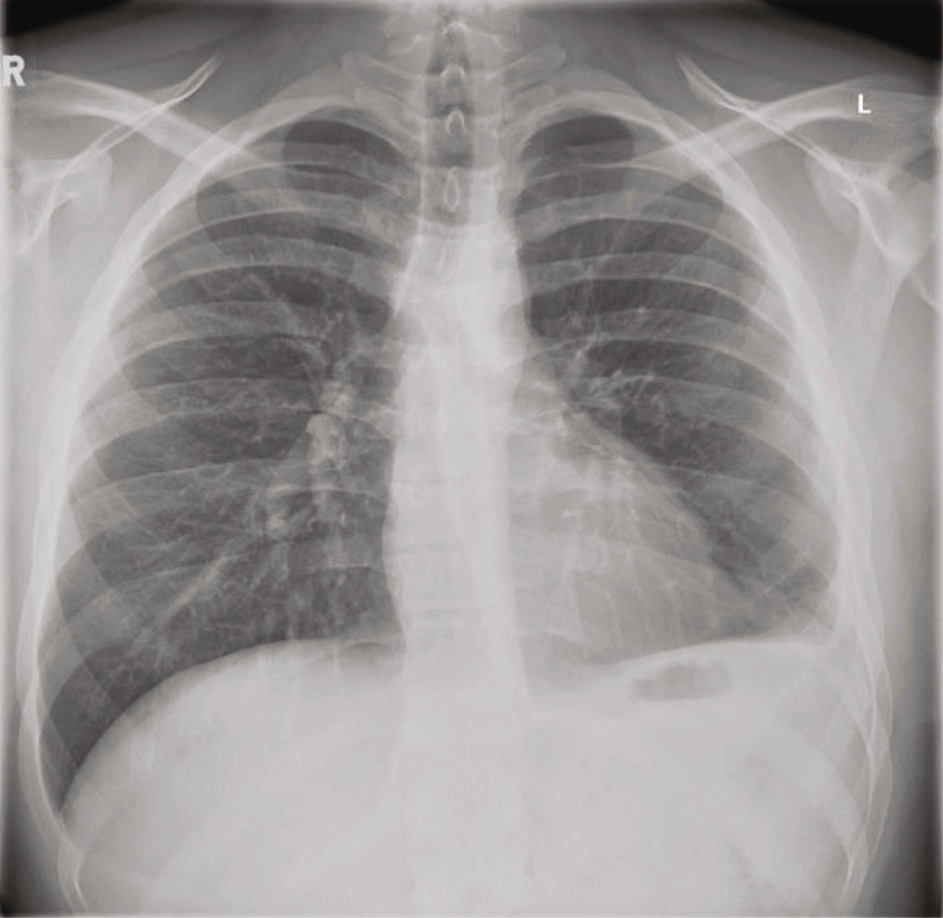
Chest X-ray taken at 2 weeks postdischarge showing clear lung fields and minimal blunting of the left costophrenic angle.

The ICD was removed three days later after 24 hours of minimal output and chest X-ray (CXR) showing a small volume of residual effusion. A day later, the patient developed a new fever and rising inflammatory markers. A repeat chest CT revealed a moderate volume haemothorax (Figure 1(b)). Thoracic ultrasound (TUS) identified an organized pleural space resembling haematoma with minimal pleural effusion. Given concerns over an evolving empyema in a retained haemothorax, co-amoxiclav was changed to piperacillin/tazobactam, and a 12Fr ICD was inserted under ultrasound guidance into the complex pleural effusion. This initially drained 300 mL of red-brown fluid (degraded haemoglobin) followed by a further 800 mL after a single dose of 10 mg intrapleural alteplase diluted in 10 mL of normal saline. CXR the following day showed an increased in the size of the pleural effusion. TUS however demonstrated a finely organized collection with minimal free flowing fluid. Together with a stable haemoglobin and minimal ICD output, it was felt that the changes were more likely due to an evolving empyema than recurrent pleural haemorrhage. A further two doses of intrapleural fibrinolytic therapy (10 mg alteplase and 5 mg dornase alfa) were administered resulting in a further drainage of 2000 mL of red-brown fluid. Subsequent chest CT showed a small residual basal collection and a thin pleural rind causing tapped lung (Figure 1(c)). Meanwhile, pleural fluid returned as an exudate with no evidence of infection on pH, microscopy, or culture. As the patient wanted to avoid surgery, an attempt to clear the pleural space and breakdown the pleural rind was made by inserting a second ICD into the basal collection, administering a further dose of 10 mg intrapleural alteplase, and placing the ICD on 4 kPa wall suction. Chest CT performed four days later showed significant improvement with minimal residual fluid and near-complete reexpansion of the trapped lung (Figure 1(d)). Both ICDs were removed, and the patient was discharged the next day. Haemoglobin remained stable throughout the admission, and no blood transfusions were required (Figure 2). On follow-up two weeks later, the patient had made a full recovery with CXR demonstrating minimal blunting of the left costophrenic angle (Figure 3).

## 3. Discussion

Haemothorax is defined as a collection of blood within the pleural space and is often the result of sharp or blunt trauma to the chest. The exact incidence of haemothorax is unclear and is estimated to be around 300,000 cases annually in the United States [1]. The initial management of haemothorax includes prompt resuscitation and drainage, usually with a large calibre (>28Fr) ICD as this allows some clots to be evacuated [2]. Drainage allows expansion of the underlying lung and permits accurate assessment of the rate of blood loss, a key factor in the deciding if early surgical intervention is required [3]. Clotted or retained haemothorax is a known complication of haemothorax and occurs in up to 20% of traumatic haemothorax [4]. Left untreated, retained haemothorax may resorb over time, become infected, or progress to fibrothorax. Studies suggest that roughly one in four retained haemothorax evolves into empyema and that evidence of angiofibroblastic proliferation, the precursor to fibrothorax, is present by day 7, as seen in this case [5]. For these reasons, it is critical that retained haemothorax be evacuated promptly.

Video-assisted thoracoscopy surgery (VATS) is the treatment of choice for retained haemothorax however requires patients to be able to tolerate general anaesthesia and single lung ventilation [6]. Furthermore, up to 20% of VATS procedures require conversion to thoracotomy, increasing postoperative morbidity and the risk of neuropathic pain [7]. Intrapleural fibrinolytic therapy (IPFT) has been shown to be effective in the management of complex parapneumonic effusions and empyema, decreasing the need for surgical intervention [8]. Its role in retained haemothorax, however, remains unclear with studies reporting mixed results when compared to VATS [9, 10].

The patient in our case had trapped lung secondary to retained haemothorax in the context of penetrating chest wall trauma. Although there were initial concerns over superimposed empyema, this was subsequently thought to be unlikely based on the appearance and analysis of the pleural fluid. Given his age and lack of comorbidities, our patient would be an ideal candidate for VATS. However, due to his personal preference of wanting to avoid surgery, IPFT was utilised as the next best management strategy with huge success. IPFT is unlikely to have any effect on chronic pleural peel as these are mainly composed of collagen in contrast to the high fibrin content in its early phases of development [3]. We hypothesize that the early and aggressive use of IPFT in our case contributed to the success in breaking down the pleural rind and facilitating lung reexpansion.

Bleeding post IPFT is a feared complication that fortunately rarely occurs [11]. The use of IPFT in individuals with high bleeding risk (e.g., acute bleed, dysregulated clotting, and recent major surgery) has not been well studied, and thus, the risk remains uncertain. In our case of acute traumatic haemothorax, we did not observe any decline in haemoglobin associated with the repeated administration of IPFT.

In summary, this case highlights the feasibility of using IPFT in the management of trapped lung associated with acute traumatic retained haemothorax as an alternative to surgery.

## Abbreviations

CT: Computed tomography
CXR: Chest X-ray
ICD: Intercostal drain
IPFT: Intrapleural fibrinolytic therapy
TUS: Thoracic ultrasound
VATS: Video-assisted thoracoscopy surgery.
